# *“I just have to take it”* – patient safety in acute care: perspectives and experiences of patients with chronic kidney disease

**DOI:** 10.1186/s12913-019-4014-4

**Published:** 2019-03-28

**Authors:** Lucia New, Donna Goodridge, Joanne Kappel, Gary Groot, Roy Dobson

**Affiliations:** 1 College of Medicine Health Sciences Program, University of Saskatchewan, Saskatoon, SK Canada; 2 Department of Medicine, College of Medicine, University of Saskatchewan, Room 543 Ellis Hall, 108 Hospital Drive, Saskatoon, SK S7N 0W8 Canada; 3 Department of Medicine, University of Saskatchewan, Saskatoon, Saskatchewan Canada; 4 College of Pharmacy and Nutrition, University of Saskatchewan, Saskatoon, SK Canada

**Keywords:** Chronic kidney disease, Safety, Qualitative research, Safety incident reporting, Patient involvement

## Abstract

**Background:**

Frequent hospitalizations and dependency on technology and providers place individuals with chronic kidney disease (CKD) at high risk for multiple safety events. Threats to their safety may be physical, emotional, or psychological. This study sought to explore patient safety from the perspectives and experiences of patients with CKD in acute care settings, and to describe willingness to report incidents utilizing an existing safety reporting system.

**Methods:**

This study was conducted using a qualitative interpretive descriptive approach. Face to face interviews were conducted with 30 participants at their bedside during their current hospital admission. The majority of the participants were 50 years or older, of which 75% had a confirmed diagnosis of end stage renal disease with the remainder at stages 3 or 4 of CKD. Eighty percent of the participants were either on hemo- or peritoneal dialysis.

**Results:**

Participants expected to receive safe care, to be taken care of, and to be cared for. Safety threats included: sharing a room with patients who were on precautions; lack of cleanliness; and roommates perceived to be threatening. The concepts of being taken *care of* and being *cared for* constituted the safety threats identified within the interpersonal environment. Participants felt taken *care of* when their physical needs are met and *cared for* when their psychological and emotional needs are met. There was a general lack of awareness of the presence of a safety reporting system that was to be accessible to patients and families by telephone. There was also an overall unwillingness to report perceived safety incidents, although participants did distinguish between speaking up and reporting.

**Conclusions:**

A key finding was the unwillingness to report incidents using the safety reporting system. Fear of reprisals was the most significant reporting impediment expressed. Actively inviting patients to speak up may be more effective when combined with a psychologically safe environment in order to encourage the involvement of patients in patient safety. System-wide organizational changes may be necessary to mitigate emotional and physical harm for this client population.

**Electronic supplementary material:**

The online version of this article (10.1186/s12913-019-4014-4) contains supplementary material, which is available to authorized users.

## Background

Patients place significant trust in the health care system, expecting that they will be safe-guarded from harm while they are being cared for in the hospital. When negative experiences or trauma resulting from safety incidents occur, this trust may be jeopardized. Patient safety is the absence of preventable harm during a patient’s health care encounter [1] and includes being treated equitably and with dignity [2]. Threats to safety may be physical, emotional, or psychological. [3] Emotional harm [4] may be the result of being treated with a lack of dignity or respect, being subjected to poor communication, being in an environment that deters an individual from speaking up or asking for help, or from the design of the health care system.

Safety is a critically important aspect of health care for persons living with chronic kidney disease (CKD). Frequent hospitalizations and dependency on technology and providers to stay alive place these individuals at high risk for multiple safety events [5–9]. Better understanding of the safety-related experiences of this patient population can contribute to improvements in quality of care.

CKD affects 10–15% of the adult population worldwide and is associated with poor quality of life, increased risk for cardiovascular disease, and reduced life expectancy [10]. CKD is associated with age-related kidney function decline accelerated by diabetes, obesity, cardiovascular disease, and hypertension [11, 12]. Severity of CKD is classified into stages according to the rate of glomerular filtration as well as the presence of albumin in the urine [13]. The progression from CKD to end-stage-renal-disease (ESRD) occurs when individuals are on long term renal replacement therapy such as dialysis, or require a renal transplant. A recent report from the Canadian Institute for Health Information (CIHI) [14] noted that there were 20,690 patients on dialysis in Canada in 2014, representing a 30% increase over the previous ten years. Within Indigenous populations, the rate of progression to ESRD is three times higher than that of the general population living with renal disease [15].

Many patients with CKD are capable of self-managing their health condition, with appropriate medical and personal support systems and a high level of health literacy. Nonetheless, due to the complexity of the disease, self-management can be a challenge for some people [16, 17]. Individuals living with CKD often have serious comorbidities, may require the need for invasive treatments such as dialysis and an increased likelihood of frequent hospitalizations [5]. Safety incidents associated with hospitalization, such as nosocomial infections, missed laboratory results, and medication errors, may predispose these patients to further kidney damage and increased lengths of stay [6–9, 18, 19].

Although patients with CKD are frequently admitted to hospital, relatively little is known about the ways in which they participate in safety initiatives. This study sought to explore patient safety from the perspectives and experiences of patients with CKD in acute care settings, and to describe willingness to report incidents utilizing an existing safety reporting system.

## Methods

A qualitative interpretive descriptive approach [20–22] informed an understanding of patients with CKD and their experiences with patient safety while they are hospitalized. Interpretive description acknowledges that people’s realities are socially constructed, subjective and shaped by their life experiences. Participants’ responses were gathered through face to face interviews at their bedside during their current hospital admission.

### Participants and setting

Purposive sampling was used to recruit participants who are typical of the population intended for this study [23]. Intentionally recruiting inpatients with CKD who may have had multiple encounters with the hospital system enabled meaningful contribution toward the goal of this study [24]. A list of eligible patients was obtained by attending weekly multidisciplinary inpatient nephrology rounds. Eligible participants were then personally recruited by the researcher to the study.

Eligibility criteria included patients: over 18 years of age at CKD stages 3, 4 or 5 (end stage renal disease [ESRD]) who are currently on dialysis or conservative treatment; admitted to hospital with peripheral vascular disease, sepsis; infections of extremities; or infections of peritoneal or hemodialysis catheters; able to provide informed consent; and participate in face to face interviews. Non-English speaking patients were eligible if an interpreter was available. Patients with CKD whose current length of stay was less than 24 h, admitted into the observation or intensive care unit, or whose current hospitalization was a result of renal transplant surgery were ineligible to participate.

The study was conducted at an urban, tertiary care center located in a core neighborhood, on one surgical and three medical units. While patients requiring isolation typically are assigned single rooms, demand often exceeds supply, necessitating the use of either two-, or four-bed rooms. Subsequently, patients with CKD may be in a room with individuals who are on isolation precautions. At the time of the study, a telephone safety response system was in place, accessible to staff, patients and families. Posters displaying the principles of the provincial safety alert initiative (stop-alert; assess-fix-eliminate; escalate and report) and the phone number to call were to be located in patient care areas and visible to patients and families. Patients and families were able to report situations that were of safety concern to them by calling from an in-house phone or from an external line. While not explicitly stated in the organizational policy, all patient reported safety incidents required follow-up.

### Data collection

Thirty participants were interviewed at their hospital bedside, ensuring as much privacy as possible, between October, 2017 and February, 2018. Data were collected using an open-ended interview guide (Additional file 1), comprised of six items, that was adapted from previous studies examining patient and family perspectives on safety engagement [25, 26]. In interviews averaging one hour in length, participants discussed their experiences and/or their concerns with safety. Family members who were present were invited to add to the interview if they chose to. In order to frame their thinking around patient safety, participants were initially requested to recall any personal, family or friends’ experiences with potential or actual harm during hospital admissions. All interview responses were audio recorded with permission and transcribed verbatim.

Additional strategies were employed to ensure rigour. Interviews were conducted by a single researcher using the same interview guide consistently. Initial coding was performed by two researchers. As well, NVivo® software assisted with data analysis. An audit trail was maintained throughout the study, including the recording of field notes after each interview.

### Ethical considerations

Ethics approval was obtained from the University Behavioural Ethics Board (17–300). An explanation of the study purpose and informed consent was obtained prior to the start of each interview. Participants were apprised of the option of discontinuing at any time during the study.

### Data analysis

Transcribed data were initially read and coded independently by the researchers (DG and LN) for a preliminary analysis. Data were subsequently entered into QSR NVivo® Version 11 for further coding and thematic analysis. Iterative coding was followed by systematically working through data segments to identify patterns. Data collection and analysis occurred concurrently, enabling a more comprehensive understanding of the phenomena or issues. Analysis of participant responses after each interview informed subsequent interviews. Questions were revised to further explore emerging themes. Immersion in the data by repeatedly reading and reflecting on transcripts and audio recordings assisted in the interpretation of participant experiences of safety in the hospital. Data were examined iteratively for possible interpretations and in relation to the research questions. Through this process, an understanding of what constitutes as safe care for participants in this study emerged.

## Results

Participant characteristics are described in Table 1. The majority of participants (73.3%) were 50 years or older, with the majority having an education level of grade 10 and higher. Approximately half of the participants self-declared as Indigenous Canadians. The majority were either retired or currently not working and lived with comorbidities of hypertension, diabetes, or peripheral vascular disease. Almost 75% of the patients in this study had a confirmed diagnosis of ESRD, while the remainder were at CKD stages 3b or 4. The majority of participants (60%) had been hospitalized in an acute care environment more than five times within the last five years.

**Table 1:** Participant Characteristics (*N* = 30)

Characteristic	n (%)
Sex	
Male	16 (53.3)
Female	14 (46.7)
Age	
≤ 50 years	8 (26.7))
≥ 51 years	22 (73.3)
Self-declared Ethnicity	
Non-Indigenous Canadian	16 (53.3)
Indigenous Canadian	14 (46.7)
Education Level	
< Gr. 9	6 (20)
> Gr. 10	24 (80)
Length of CKD	
≤ 5 years	19 (63.3)
≥ 6 years	11 (36.7)
Stage of CKD	
3	3 (10)
4	5 (16.7)
5 (ESRD)	22 (73.3)
Type of Dialysis	
Hemodialysis	17 (56.7)
Peritoneal	6 (20.0)
Not currently on dialysis	7 (23.3)
Comorbidities	
Present (diabetes, hypertension, PVD)	25 (83.3)
Absent	5 (16.7)
Number of hospitalizations in past 5 years	
< 5 times	12 (40.0)
5–10 times	6 (20.0)
> 10 times	12 (40.0)
Admitting Diagnosis	
Infection of lower extremities	9 (30.0)
Infection to dialysis catheters	3 (10.0)
Other (HF, pancreatitis, back pain, abd pain, gastroparesis, pancreatitis, post-op fracture)	18 (60.0)
Length of Stay	
≤ 15 days	21 (70.0)
≥ 16 days	9 (30.0)

Despite the frequent encounters with hospital care, many of the participants were not able to recall significant experiences of harm with previous hospitalizations. Some participants described safety incidents that had been experienced by family members or acquaintances. Others indicated they had only heard of incidents through the news media. The most common past potential harm personally experienced by a few of the participants involved medications. Participants described instances where they would have been administered medications prescribed for other patients, were it not for their knowledge regarding their medication regimen and their willingness to ask questions of the nursing staff.

Concerns about safety risks and observations of care were similar regardless of whether participants were at earlier stages of CKD or in ESRD. Several participants who were on dialysis voluntarily compared the physical and interpersonal care environments between the hospital and dialysis units. A greater number of safety concerns were voiced about the inpatient hospital experiences. Situations or interactions raised by participants were mostly non-technical and related to service delivery. Conversely, technical aspects of safety were identified only after prompting, suggesting that they are currently embedded into routine patient care. These include practices such as: medication administration (identification checks of patients); access of intravenous lines, hemodialysis vascular catheters or arteriovenous fistulas (antiseptic cleansing of ports prior to access); as well as handwashing before and after patient care. Participant responses are discussed under the main themes of: receiving safe care; expecting to be taken care *of*; expecting to be cared *for*; and reporting safety concerns.

### Receiving safe care

Participants expressed being concerned for their personal safety from elements in the physical environment, including: being in a room with patients who were isolated for an infection; lack of cleanliness; roommates perceived to be threatening; and other patients and visitors. Comments from participants are located in Table 2.

**Table 2:** Receiving safe care

Subthemes	Participant comments
Sharing a room with patients on isolation	*“Sometimes they put people in gowns and [they] have these things that shouldn’t be transmitted to other people. They put them in with people that don’t have that and they, they say that’s a good enough divide.” (P21)*
Roommates perceived to be threatening	*“Yeah, like the people they put me in a room here, the last time I was in the hospital they put me in a room with a guy that wasn’t all there and it scared me … finally I made myself get up … and went to sleep in those chairs over there.” (P5)*
Lack of cleanliness	*“It’s not very clean in here for being a hospital … how can you get good, safe health when the insides of the rooms look like hell.” (P6)*
Other patients and visitors	“*… Like after dark I won’t go outside out here. Unless I have somebody with me or whatever. Cause there’s too much riffraff...” (P18, family member)*

#### Sharing a room with patients on isolation

Participants were worried that they would be at risk for contracting another infection when sharing a room with a patient on isolation, due to their perception of an already compromised immune system. They observed that good infection control practices were not consistently followed. Subsequently, some participants felt that they had to be vigilant and monitor for any breaches in practice. The effectiveness of curtains as an infection control barrier was also criticized by several of the participants.

#### Lack of cleanliness

Some participants were worried that the lack of cleanliness would be a detriment to their compromised health status, placing them at risk for contracting hospital acquired infections They described taking on the responsibility to maintain the cleanliness of their own space, due to their concern.

#### Roommates perceived to be threatening

Being in a room with a patient they perceived to be a threat caused participants to be worried for their personal safety. Some participants were also concerned about threats to the safety of the other patients and staff from patients or visitors who were exhibiting threatening and disturbing behaviors.

#### Other patients and people

Some participants spoke of the distress they felt listening to other patients yell, cry out, or moan loudly. A few of the participants were apprehensive when unfamiliar people came and sat in their rooms and indicated the relief they felt when nursing staff were readily available to intervene. Some participants expressed annoyance, or feeling unsettled, with people who were merely wandering around the hospital, occasionally bothering patients.

### Expecting to be taken care *of*

Patients expect their basic physical, psychological, and emotional needs to be met through interactions with care providers. By having these needs addressed, patients feel they are taken care of and are cared for. The concept of being taken *care of* differs from that of being *cared for*. *Care of* the patient addresses physical needs, such as being clean, having adequate pain control, and coordination of care amongst multiple providers. Physical safety is maintained when these needs are met, whereas psychological and emotional safety is addressed when patients feel *cared for* and perceive they are valued as a person rather than just a patient.

Being hospitalized meant that participants were dependent on care providers for having their basic physical needs met. The level of dependency varied with participants’ health status. Communication amongst multiple providers involved in their care, communication between providers and participants, and delays in care were some of the concerns expressed. Observations from participant related to being taken *care of* are in Table 3.

**Table 3:** Expecting to be taken care *of*

Subthemes	Participant comments
Communication amongst providers	*“Your nurses change every day. Your doctors change every week. … and just communication needs to be better for patient safety for sure.” (P21 family member)*
Communication between providers and participants	*“I don’t feel safe because what is, something is going on, they are not talking to me if nothing is going on but I would like to know either way and I’ve asked.” (P9)*
Delays in care	*“I usually have to ask to get rolled over or something.” (P22)* *“Well sometimes they take too long to come, that’s a big, big thing. I’ll be sitting in pain … and I was always wanting to use the washroom and … I have to force myself to get up and do it for myself …*” *(P5)*

#### Communication amongst providers

Depending on the reason for admission, patients with CKD may be under the care of two or more physicians during the length of their hospital stay. Participants brought up concerns about the lack of communication within the physician group, between physicians and nurses, as well as within the nursing group itself.

#### Communication between providers and participants

Some participants expressed frustration with the irregular amount of updated information provided to them. One participant, whose encounters with the health care system dated back to childhood, observed that “*communication throughout the system is hard on patients*” *(P21)*. While some participants were able to clarify information with physicians, others spoke of being discouraged from asking questions. Some believed their level of knowledge was inferior to physicians and therefore did not feel they were in a position to ask questions.

#### Delays in care

Many participants expressed frustration with the delayed response to their calls for assistance. However, they were generally forgiving of these delays and attributed them to workload and staffing issues. Some spoke of being “fed up” while waiting for care providers to return as promised. Others voiced their indignation with the lengthy wait for assistance with basic care, such as toileting or bathing.

### Expecting to be cared *for*

Patients’ perceptions of whether they are cared for, or cared about, are impacted by the nature and quality of patient-provider relationships and interactions. Psychological and emotional support is characterized by expressions of empathy, compassion, dignity and respect. While several participants talked about positive interactions with care providers, there were also narratives of grievous experiences that threatened participants’ emotional safety. Trust in their providers was also brought up by some participants as contributing to their feeling of safety. Table 4 contains participant comments about being *cared for*.

**Table 4:** Expecting to be cared *for*

Subthemes	Participant comments
Interactions with health care providers.	*“So you begin to see which nurses you feel comfortable with … because they treat you with a little bit of dignity and a little bit of empathy, compassion.” (P26)* *“They’re all very communicative. And they answer your questions. You know they always take the time.” (P18 family member)*
Trust	*“No, I leave it up to the professionals to do what they do, what they think is right.” (P13)* *“Well, I’m laying here and I trust my nurses when I come in. They tell me what they’re going to give me.” (P16)*

#### Interactions with health care providers

Both negative and positive experiences with the care and overall interactions with health care providers were described. Most participants viewed their care to be generally positive but there were others who felt that the demeanor and attitudes of the care providers could be improved. There were participants who experienced their concerns or questions being dismissed, or perceived they were not being listened to. Participants talked faster and in a louder, higher tone of voice when recounting exchanges perceived to be dismissive or disrespectful. They described verbal and non-verbal exchanges as well as observations of body language that caused them to feel angry or frustrated. A feeling of resignation was expressed by one of the participants: *“I’m just like everyone who goes with the flow. There’s nothing you can do about it” (P20).* For some individuals, care was perceived to be impersonal, in part because the staff seemed to be constantly in a rush. Conversely*,* some participants talked about the positive relationships developed with care providers over the course of their health care encounters and the psychological and emotional support experienced.

#### Trust

While some participants stated that they had no expectations of care while in the hospital, several indicated that they simply expected to be “looked after” and trusted providers to do so. Participants commented on the tendency to rely on nursing staff more than other provider groups. They felt that the majority of their interactions occur mostly with the nursing staff. Therefore, that group was perceived to have a better grasp of the patient’s condition and needs. Despite the trust expressed, some participants believed it was important for family members to be present, to advocate for the patient’s care and safety.

### Reporting safety concerns

Subthemes included: participants’ willingness to speak up; responses received from care providers; awareness (or lack) of the safety reporting line; and their willingness to report safety concerns using the telephone line. Table 5 provides insights from participants on the reporting of safety concerns.

**Table 5:** Reporting Safety Concerns

Subthemes	Participant comments
Speaking up	“*… it depends on the health of the patient. When you’re weak and out of it, not even thinking straight, it’s pretty hard for you to do it” (P2)*
Reactions to questions asked	*“Don’t always get the answers and don’t always get the response in terms of like it’s just like they don’t want to tell you. Some are genuinely responsive, others like I say don’t bother us, you’ve been taking this for the last, you know, the same thing.” (P2)*
Awareness of safety line	“*This is the first I’ve ever heard of it … I mean I’ve never heard of it all these years I’ve been in the hospital.” (P12)*
Using the Safety Line	
Lack of trust regarding response to reporting	*“If it’s not written down on paper, nobody will know anything about it … Because you would never know if anything was done. You’d know, you reported but you would never know if anything was done about it.” (P10)*
Fear of reprisal	*“I just don’t really like causing a lot of problems because the next thing you know, these are the same people that have to give me needles and they are poking me …*” *(P22)* *“Make me feel like I got a little more security, but if they ever found out that I did that …*. *Oh, they would treat me like dirt, I bet you.” (P14)*
Added stress with reporting	*“I don’t need extra stress kind of thing. … Where do I report it to in the first place … Sometimes causing waves is probably more stressful in my life than not causing waves either.” (P18, family member)*

#### Speaking up

Several participants indicated that they would speak up about concerns experienced in their day-to-day interactions regardless of the response anticipated. One participant reported his willingness to speak up was strongly encouraged by a family member who was a health care provider. Another participant, who had had experiences with safety incidents with previous hospitalizations, commented *“Well I definitely ask what kind of, what am I given.” (P2)*. Situations in which participants had spoken up primarily involved medications. Some participants brought up occasions when they were provoked into responding with anger or frustration. Several participants believed that while it is important to advocate for oneself, it was not always possible due to the health status at the time, *“Patients are typically too sick and too scared.” (P17)*.

#### Responses from care providers

Mixed responses were experienced by participants who participated in their own care by speaking up, asking questions or reminding staff about personal protective equipment. For some participants, speaking up was important for self-protection, “*… I said okay, you know I’m just … looking out for myself” (P2)*. Responses ranged from expressions of appreciation for reminders, providers welcoming of participants’ questions, to body language or facial expressions that conveyed annoyance. Despite the negative reception to their input, some participants were adamant about the importance in self-advocating for their care and safety.

#### Awareness of the safety line

Very few of the participants were aware of the existence of the safety telephone line that was available to patients or families for reporting concerns. A recommendation was made by a family member to have the information posted, increasing the visibility to patients and families. Participants did not have alternative strategies for reporting safety concerns and indicated they did not know who they would report to. Some participants expressed that they would voice their concerns to the nursing staff or the head nurse first, and then further up the chain of communication as necessary.

#### Using the safety line

The majority of participants who learned about the availability of the safety line during the interview indicated they would likely not use it, mostly due to fear of reprisals from staff. The fact that the clinical staff are the ones who will continue to provide care to them prevented some participants from reporting. Some participants talked about the level of educational knowledge and experience providers have in comparison to their own. In one participant’s words: *“I cannot know what the doctor is doing, if it’s right or not. I just have to take it” (P17)*. Another reason offered was the lack of energy for anything beyond looking after themselves while sick in the hospital or in the case of a family member, providing support for their spouse. Some participants did not want to cause trouble for the staff, particularly the ones perceived to be kind and caring. There was also an overall lack of trust that concerns reported would be responded to in a meaningful way.

## Discussion

Findings from this study have demonstrated that in spite of the availability of a safety line and the recognition that their safety in hospital was jeopardized, participants were reluctant to report safety concerns. Consistent with other studies [27–32], the lack of awareness of the safety reporting line, fear of repercussions, poor health, and dependence on providers for current and future care limited the willingness of participants to report concerns. Concerns within the interpersonal and physical environments were recounted by participants in this study. Within the interpersonal environment, participants described situations and interactions related to perceptions of being taken *care of* and being *cared for*.

Despite the institutional policy of having a reporting system accessible to patients and families, signs displaying the access information were not visible in any of the patient care areas. The contribution to patient safety when patients are able to give voice to their concerns [32, 33] and their willingness to do so when actively encouraged [30, 34] is well supported. Awareness of access information for reporting concerns may be one way of encouraging patients to engage in safety reporting. The absence of this visual cue for reporting concerns was a barrier to engaging participants in patient safety. Putting the onus on patients to seek the mechanism of reporting means the opportunities to improve patient care may be lost. However, while the lack of awareness created a barrier, a more concerning obstacle was the perception of power differential between care providers and patients.

A belief in the superior knowledge of the care providers compared to their own limited knowledge resulted in participants feeling they were not in a position to question, or contribute to, decisions made by providers. The notion of power imbalance has been found by other studies involving the inpatient population [35, 36]. Imbalance of power was not experienced by all participants as some did verbalize the willingness to report concerns, recognizing that doing so may have implications on their care and treatment. Nonetheless, they felt it was important to advocate for themselves. Past exposure to safety events, encouragement from family members, having a higher level of education, and a previous professional occupation were all contributing factors for individuals who expressed the need to speak up.

Participants differentiated between “speaking up” and formally reporting safety concerns. For some participants, speaking up meant asking questions and for others, it meant asserting their right to be treated with respect, to be given information regarding care plans, or to have their basic care needs met. Whereas most participants were able to “speak up” at the point of care, formal reporting of safety concerns using a reporting system was intimidating due to perceived possible negative consequences. In order for patients to voice their opinions or concerns without fear of consequences, a psychologically safe environment is required. [36–38] Participants indicated that such an environment was absent and talked about feeling vulnerable, helpless, and incapable of advocating for themselves. The inability to speak up and fear of reporting may further reinforce participants’ feelings of powerlessness and increase their dependency on care providers. A psychologically unsafe environment is counterintuitive to the concept of patient-centered care and may negatively impact the health outcomes of patients [36].

Similar to other studies [39, 40], narratives from participants in this study were largely related to service quality and interactions with care providers. Provider actions and behaviours resulted in participant perceptions of being taken *care of* and being *cared for*. Unlike the majority of the research on patient experiences of care [41–45], a distinction is made between these two concepts in this study. While the concepts are discussed separately, there is considerable overlap between the two. Participants in this study shared positive and negative experiences with respect to being taken care of and being cared for.

Being taken *care of* includes expectations of basic needs being met, their care plan communicated with them and information-sharing by providers to occur. The majority of the participants were satisfied with their care. For the instances when needs were perceived to be unmet, or perceived harmful actions occurred, participants did not want to jeopardize their relationship with providers by speaking up and contributing to their workload and stress. Issues with communication that led to participants feeling uninformed and receiving fragmented care resulted in perceptions of increased dependency on care providers. A study by Davidson et al. described the value participants placed on the concepts of care with information (being kept informed) and involvement (involved in the plan of care) [45]. As other studies have found [27–29], whether participants chose to take on an active or passive role depended on intrapersonal factors such as: frequency of healthcare encounters; length of time an individual has been interacting with the health care system; educational level; and the encouragement received from family. The passive acceptance of being taken care of and the need to maintain relationships may indicate that the socially constructed traditional and passive role of the patient is firmly entrenched for some individuals.

Patients feel they are *cared for* through positive relationships and the interactions with providers during the hospital stay. When perceived that they are valued as a person as opposed to a mere patient, they determined their psychological and emotional well-being to be sufficiently supported. [41, 42, 44] Care received that was perceived to be person-centered resulted in participant reports of being treated as an individual. Conversely, emotional distress was caused by care perceived to be impersonal and rushed. Perceptions of being cared for and having their dignity maintained as being dependent on patient-provider interactions and provider behaviors are confirmed by previous studies [46–48]. McCabe [46] found that nurse-patient relationships that are superficial, task-focused, and non-patient centered, were a barrier against patients feeling safe, or cared for.

Several participants indicated that they felt safe because they “trust” care providers to look after them. Those who had personal experiences of questionable or unsafe care stressed the importance of being vigilant about their own safety rather than relying on care providers. Mollon [49] also found that patients developed feelings of distrust and felt unsafe when expectations of their care were not met. It was important for participants to have family present, acting as their advocate. Relatives functioned as a buffer system against potential safety incidents, shouldered the burden of needing to be vigilant, and provided support for emotional well-being. The necessity of family members to ensure patients’ safety and meeting emotional needs has been found in other studies [46, 50, 51].

Within the physical environment, participants identified threats to their physical health as well as personal safety. Participant responses were similar regardless of whether they were in earlier stages of CKD or at ESRD. In order to compare whether patient perceptions varied by stage of kidney disease, additional research adequately powered to distinguish variations between the stages will be needed. Previous studies have found CKD to be a risk factor for a greater number of patient safety incidents [5–7, 52–54] with effects deemed to be more significant given the underlying pathophysiology of the disease.

Specific to this study was the willingness of some participants to advocate for their safety by being vigilant about, and occasionally requesting, providers to change their personal protective equipment (PPE) prior to providing care to them. This finding is in contrast to research conducted by Davis and colleagues [27, 55] where participants are more willing to ask general questions about the management of their care than challenging questions such as handwashing, at the risk of offending their care providers. Similar to other studies [38], participants spoke of their vulnerable state and the physical inability to protect themselves from other patients and visitors within their physical environment at the hospital. While the risk to personal safety from other patients has also been found in another study [33], identifying a safety threat from visitors is another finding unique within this study.

### Practice implications

To date, patient safety strategies have focused primarily on physical harms, caused by technical safety infractions. Regardless of whether incidents are technical or non-technical related, all safety events can cause emotional harm of varying degree. Mitigation of emotional harm is equally essential. It is even more important that patients are intentionally invited to play a role in maintaining their safety, recognizing for some, this may be difficult or unwanted. Increasing the visibility of the safety reporting system is the first step in encouraging patient and family involvement. Another strategy may be to improve the ease of reporting such as through a mobile handheld application [56]. Furthermore, responding to patient concerns in a timely fashion may reinforce the value of reporting. Fears of retaliation may be addressed by the establishment of a psychologically safe environment. The involvement of patients in patient safety may be more effective when patients are invited to speak up in and feel safe to do so in the presence of such an environment. It may be worthwhile to ask for patient feedback on a real-time basis, through face to face interviews as patients’ perceptions of patient safety may differ from those of care providers. For the participants in this study, the concept of safety related to non-technical aspects of safety such as: interactions with providers; quality of care; and being treated as a person, versus the technical aspects which are the focus of healthcare organizations regarding patient safety strategies. Nonetheless, system-wide safety improvement strategies need to be designed and implemented to protect the safety of this vulnerable population.

One strategy may be to establish safety leaders, such as patient safety officers, as part of multidisciplinary teams providing care. The overall goal is to drive, and support, safety transformation processes by engaging care providers at the frontline, clinical leaders, and administrative leaders. A key responsibility of the safety officer is to bring patient and family input into operation of healthcare organizations [57]. Another solution may be to redesign health care delivery systems for coordination and integration of care, such as accountable care units, which have proven to result in better quality of care for patients [58, 59]. Regardless of whether improvement strategies involve the presence of patient safety leaders, system redesign or even the establishment of WalkRounds™ [60], patient safety outcome must be evaluated.

### Limitations

Findings from this study represent a select sample of participants from one tertiary hospital. As such, results may not be applicable to other tertiary centers or other patient populations as part of the wider patient safety component of quality improvement strategy. Purposive sampling was used to recruit participants. Therefore, voices are missing from patients with CKD who may be at a higher risk for safety events, such as individuals whose first language is not English. Although the perspectives of the participants in this study were similar, patients with ESRD may have a different perspective on safety risks than ones at earlier stages of CKD, due to possibly more healthcare encounters and risks accompanying dialysis treatments. Exploring potential differences in experiences and perspectives between the two subsets of this particular patient population would contribute to the evidence base. Additionally, expanding the research to include patients with other chronic illnesses and from multiple sites may provide a more informative picture of factors that promote or hinder patient engagement with safety. Subsequent information may be more useful to the practice setting, specifically with safety promotion and harm prevention strategies.

## Conclusion

A key finding in this study was the unwillingness of participants to report incidents using the safety reporting system currently in place. One of the major barriers is the lack of awareness of the reporting system. Participants also expressed a lack of knowledge regarding whom to report to. However, it may be the fear of retaliation that poses a more significant impediment to reporting of safety incidents. In order to improve care from patient feedback, these barriers will need to be addressed. The establishment of a psychologically safe environment is an essential first step in this process. Overall, system-wide changes at an organizational level, with engagement of front-line care providers, clinical and administrative leaders, as well as patients and families are needed.

Participants were able to identify safety threats from the physical and interpersonal care environments that have the potential to cause physical and emotional harm. Strategies to address patient safety must include physical as well as emotional safety. Patients with CKD may already possess the knowledge to self-manage their condition and may welcome the opportunity to be involved in their safety in the hospital setting. Preparing them for taking on an active role must begin while they are in a healthy state, versus while they are in a vulnerable state during their hospital stay, so that they are able to gradually incorporate them into everyday behaviour and action. At the same time, the culture must change so that what patients are being asked to take on is positively received by health care providers [32, 33].

## Additional file

Additional file 1: Patient safety in acute care-Interview Guide. Interview questions for participants (PDF 59 kb)

## Abbreviations

CIHI: Canadian Institute of Health Information
CKD: Chronic kidney disease
ESRD: End stage renal disease
