# Supplementary material for: “I just have to take it” – patient safety in acute care: perspectives and experiences of patients with chronic kidney disease
Source: BMC Health Serv Res. 2019 Mar 28;19:199. doi: 10.1186/s12913-019-4014-4 (PMC6437896; doi:10.1186/s12913-019-4014-4)
Supplement: Supplementary file 1 — Patient safety in acute care-Interview Guide. Interview questions for participants (PDF 59 kb) [file 12913_2019_4014_MOESM1_ESM.pdf]

**Patient Safety in Acute Care-Interview Guide**

1. There have been reports in the news about things going wrong in the hospital that caused harm to patients. What are some examples you've heard about – either from the news or from friends and family members?
   **Prompts**: what have you heard about patients getting the wrong medications, infections from surgeries, or injuries from falls? Any others?
2. While you've been in the hospital, what are some things you've seen that have made you wonder about whether safety is an issue?
   **Prompts**: spills on the floor, equipment not working well, or bloodwork or tests being missed?
3. Was there ever a time when you felt unsafe or had questions about your care?
   **Prompt:** Did you let anyone know? If so, who was it?
   **Prompt:** What changed after you had told someone about your concerns?
   **Prompt:** What were you hoping they would do to help with your situation?
4. When patients and families have concerns about their safety, there is a safety alert line they can call – are you aware of it, or have you ever used it?
   **Prompt**: now that you are aware of it – would you use it?
   **Prompt**: what would make you use it?
   **Prompt**: what would prevent you from using it?
   **Prompt**: what is the response you would expect to get when you report your concern?
5. It takes the effort of a lot of people to make sure that patients are safe while they are in the hospital. What are the things the staff do to help you feel safe? What other things do you feel they should do?
   **Prompts**: handwashing, checking to ensure correct patient
6. What are some things you can do yourself to feel safe?
   **Prompts**: reminders to staff about handwashing, making sure staff clean with alcohol before lines are accessed, knowing lab work results
   **Prompt**: How might doing these things help you to feel safe?
7. If you were to give a passing grade to a hospital in terms of how well it ensures that patients are safe, what are some actions or practices you would use for coming up with a mark?
